# Development and Implementation of an AI Tutor To Scaffold Problem-Solving in Introductory Chemistry

**DOI:** 10.1021/acs.jchemed.6c00039

**Published:** 2026-09-23

**Authors:** Karen D. Wang, Joshua T. Arens, Chinat Yu, Jordyn N. Smith, Hannah Bartels, Mercy Haub, Fun Man Fung, Nick Haber, Shima Salehi, Jennifer Schwartz Poehlmann

**Affiliations:** † School of Information, San José State University, San Jose, California 95192, United States; ‡ Department of Chemistry, 6429 Stanford University, Stanford, California 94305, United States; ¶ Graduate School of Education, Stanford University, Stanford, California 94305, United States; § School of Chemistry, 8797 University College Dublin, Dublin D04 V1W8, Ireland

**Keywords:** First-Year Undergraduate/General, Internet/Web-Based Learning, Problem Solving/Decision Making, Applications of Artificial Intelligence

## Abstract

Developing problem-solving competency is a central goal of undergraduate chemistry education, yet many students struggle to engage productively with complex, multistep problems. At the same time, the rapid adoption of large language model (LLM)–based tools by students in STEM courses raises concerns about over-reliance and uncritical use, potentially undermining learning. In this work, we present the design, implementation, and evaluation of STEPS (STEM Tutor for Effective Problem Solving), an AI tutor particularly designed to guide students in creating a problem-solving plan without divulging solutions. The tutor was deployed in an introductory chemistry course at an R1 university, and its impact was examined using performance data, Likert-scale survey responses, and qualitative analysis of student reflections. Students demonstrated high performance on STEPS-assisted problems and reported strong confidence in their solutions. Survey results indicated consistently high perceptions of helpfulness. Qualitative analysis revealed that students valued the tutor’s guidance in organizing their thinking and checking reasoning without giving away solutions. These findings suggest that carefully designed, task-specific AI tutors can promote productive engagement with problem solving while mitigating risks associated with uncritical AI use in chemistry education.

## Graphical abstract

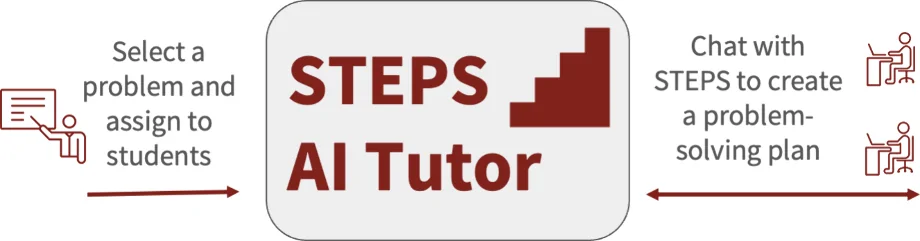

## Introduction

Developing problem-solving competency is a central goal of chemistry education.^1^ The significance of this educational goal is increasing given the rapid integration of artificial intelligence (AI) into professional and everyday life, particularly as AI systems demonstrate proficiency in handling well-structured cognitive tasks.^2^ Consequently, the ability to solve complex, unstructured problems in effective collaboration with AI tools has become an essential competency for STEM graduates.

To date, educators have explored various instructional strategies to teach problem-solving in college chemistry, including explicit metacognitive instruction for first-year college students^3^ and a systematic workflow that scaffolds students through specific phases of problem-solving in the context of an introductory chemistry course.^4^ However, chemistry education still faces major challenges in helping students develop complex problem-solving competencies. Introductory chemistry courses often rely on well-structured, textbook-style problems that strip away the complexity and ambiguity inherent in real-world scenarios. Moreover, students are typically assessed on the correctness of their final answers rather than the quality of their problem-solving processes.^5^ These oversimplifications stem from practical constraints: given large class sizes and fast-paced, content-heavy curricula, instructors face significant challenges in developing authentic complex problem scenarios and providing individualized scaffolding.^6^ Consequently, students may develop the mindset that reaching the correct answer is more important than understanding the problem-solving process,^7^ which ultimately limits their development of problem-solving competency.

It is worthwhile to examine whether and how large language models (LLMs) can help address these instructional challenges. They can help instructors create real-world, complex problems by generating case-study topics, problem statements, and mock experimental data.^8,9^ During problem solving, LLMs can provide adaptive scaffolding that guides students through the problem-solving process, thereby shifting student behavior from solution-seeking to process-oriented approaches.^10,11^ Furthermore, LLM-based tutoring systems provide students opportunities to develop productive human–AI collaboration,^12,13^ an emerging essential competency for chemistry graduates. Realizing these benefits, however, depends on careful instructional design to specify when and how LLMs are used in chemistry education, prioritizing transparency and metacognition throughout the course, and aligning tasks and assessments with authentic complex problem solving.^14^ Effective LLM integration into instruction therefore requires more than tool availability and depends on how the technology is situated within the course environment, including instructor modeling of use, clear communication of expectations for AI use, and alignment with instructional goals.^15^

Recent research indicates that college students are rapidly adopting generative AI tools such as ChatGPT as part of their academic workflows.^16^ Many students report using these tools in pedagogically productive ways, including to understand complex topics and clarify concepts.^17^ At the same time, emerging evidence suggests that students often engage with AI tools in ways that prioritize efficiency and task completion, sometimes relying on AI to directly generate solutions to problems without much critical engagement.^18,19^ Such use can further exacerbate the disconnect between traditional chemistry coursework and complex problem-solving practices. Although LLMs such as GPT-5 and Claude-3.5 Sonnet have performed well on high-school- and college-level chemistry benchmarks consisting of well-structured textbook style questions,^20,21^ they still generate incorrect solutions in a substantial portion of cases, particularly with multistep, multiconcept problems, where students struggle the most. More concerning, LLMs present incorrect solutions with the same level of confidence as correct ones, creating significant risk of misleading students and reinforcing misconceptions unless outputs are critically evaluated.^22^ Students vary widely in their ability to recognize and respond to these inaccuracies,^23^ highlighting the need for scaffolding to support AI literacy in chemistry problem solving.^24^

The approach of using AI for direct solutions may also risk incurring significant long-term cognitive costs: neurological evidence from a recent EEG study shows that LLM users exhibit different brain connectivity patterns and reduced cognitive engagement, compared to students working independently.^25^ The limitations of existing AI tools in education become even more problematic when considered alongside extensive research demonstrating that students’ productive struggles deepen their learning.^26^ In order to build effective problem-solving competency, students need opportunities to practice how to get started on complex problems, make mistakes and reflect on them, and develop their own plan that can ultimately solve the problem. Furthermore, they need to practice how to mindfully and critically leverage AI tools as a learning companion, rather than a replacement, in this process. Over-reliance on AI tools to generate immediate solutions deprives students of these critical productive struggles, ultimately undermining their development of the strategies and metacognitive skills essential for complex problem solving in the real world.

There is a clear need for AI tools designed to guide students through problem solving rather than providing solutions and to support instructors in expanding the opportunities for students to engage with complex, real-world problems in introductory STEM courses. To address this gap, we developed an LLM-powered tutor named STEPS, or STEM Tutor for Effective Problem Solving. This paper describes the design, implementation, and initial evaluation of STEPS in an introductory chemistry course and addresses the following questions:

- How can an LLM-based tutor be designed through prompt engineering to scaffold chemistry problem-solving processes?
- How can this tutor be effectively integrated into an introductory chemistry course?
- What are the educational impacts of the tutor, including student perceptions of its utility, problem-solving performance, and development of AI literacy?

## Background

### Problem Solving and Expert Decision Making

We have conducted extensive research to characterize problem-solving expertise in STEM domains.^27,28^ Our framework breaks down the problem-solving process of experts into key phases including planning, execution, and reflection, each with a set of specific decisions that must be considered and deliberately made. In the planning phase, critical decisions include determining (1) the relevant domain knowledge for solving the problem, (2) the necessary assumptions and simplifications to make the problem more tractable, (3) the information/data needed for solving the problem, and (4) methods to collect the information. Based on this framework, we designed paper-and-pencil problem-solving templates for students in college introductory physics and chemistry courses.^29,30^ We found that structured scaffolding through the template could improve student problem-solving performance when problems were appropriately complex. However, even with introductory sessions introducing the template to students, it was difficult to motivate students to work through this long, paper-based activity on their own. At the same time, our analysis of students’ problem-solving processes revealed that students struggle most with selecting and adapting appropriate domain knowledge and formulating detailed solution plans.^29^ These findings highlight the need for more targeted, adaptive support in the planning phase of chemistry problem solving, one that scaffolds both content-specific and metacognitive processes.^31^ We therefore explored how an LLM-based tutor could scaffold the planning phase and keep students engaged with it.

## Methods

### Technology Description

Existing literature on AI tutors has largely focused on general-purpose tutors with the aim of providing readily accessible resources for students to explore any topic across various disciplines.^32,33^ However, this broad approach imposes an unrealistic burden on students to verify the accuracy and appropriateness of AI-generated content, especially as current AI models remain prone to generating inaccurate information. Given that students are novices in the subjects they seek to learn, this may lead to misconceptions and reinforce misunderstanding among students. Moreover, students often lack the mental models necessary to formulate productive questions when engaging with AI tutors to support their problem-solving. Without clear guidance on how to interact effectively with these tools, students may default to asking for complete solutions rather than seeking targeted support for specific aspects of their problem-solving process.^18^ Researchers have explored approaches such as teaching students to prompt AI as a tutor rather than a solution provider^34^ and providing AI literacy workshops covering how LLMs work, prompting, and response evaluation to support productive AI use.^35^ Custom-designed AI tools represent another approach to addressing this challenge, as we demonstrate with STEPS.

In contrast to general-purpose AI tutors, the scope of STEPS is defined to scaffold problem solving in college STEM courses by guiding students through our validated problem-solving practices framework, with a particular focus on the planning phase. We have designed STEPS to function as a thought companion, mirroring how we envision LLMs will be used in future professional careers. At the same time, because its users are students who are novices in the subject material, STEPS also serves as a domain-specific tutor to answer student questions about content. Each instance of STEPS is designed for a specific problem situated in a real-world context and requires the application and integration of multiple concepts, formulas, and skills learned in class. This targeted design ensures greater alignment with specific learning goals while reducing the likelihood of LLMs generating inaccurate or pedagogically ineffective content. By adapting LLMs to ask questions corresponding to key effective problem-solving decisions and guide students through the planning process step-by-step, we created STEPS to be a tool that instructors can integrate into student learning experiences, ensuring it complements course instruction and supports student learning in a timely, targeted, and pedagogically-informed manner.

### Prompt Engineering

Our prompt-engineering process began by adapting a prompt that we had developed for a physics problem to a chemistry problem based on our validated problem-solving template.^30^ The initial chemistry prompt followed the physics prompt in its overall structure, retained the same defined roles and purpose, behavioral guidelines, and adapted the sequence of questions the LLM should pose to students. The prompt defines STEPS as a chemistry problem-solving coach that guides students through a structured question sequence: identifying relevant concepts, explaining their relevance, specifying needed information and equations, and creating a step-by-step plan. It also incorporates behavioral guidelines that prevent direct solution provision, maintain engagement, and redirect students back to the planning process when they seek complete answers or stray off-task. For each implementation in the chemistry course, we first selected or developed problems that required knowledge and skills covered in that week’s coursework. We adjusted the question sequences to target specific chemistry concepts, specified the correct answers for each question, and updated both questions and answers within the STEPS prompt. Before deployment, our teaching team, which included course instructors and undergraduate and graduate students, conducted extensive testing by adopting different student personas, including answer-seeking students, students providing minimal responses, and engaged students offering detailed explanations, to evaluate the tutor’s behavior across varied interaction styles. Based on these tests, we refined the behavioral guidelines and response patterns to handle different student approaches effectively. STEPS operated solely based on the prompt. No additional course materials were provided to or accessed by the model. Chart [Fig ch1] presents a summary of the final prompt structure. For the full prompt, see Listing S1 in the Supporting Information.

**1 ch1:**
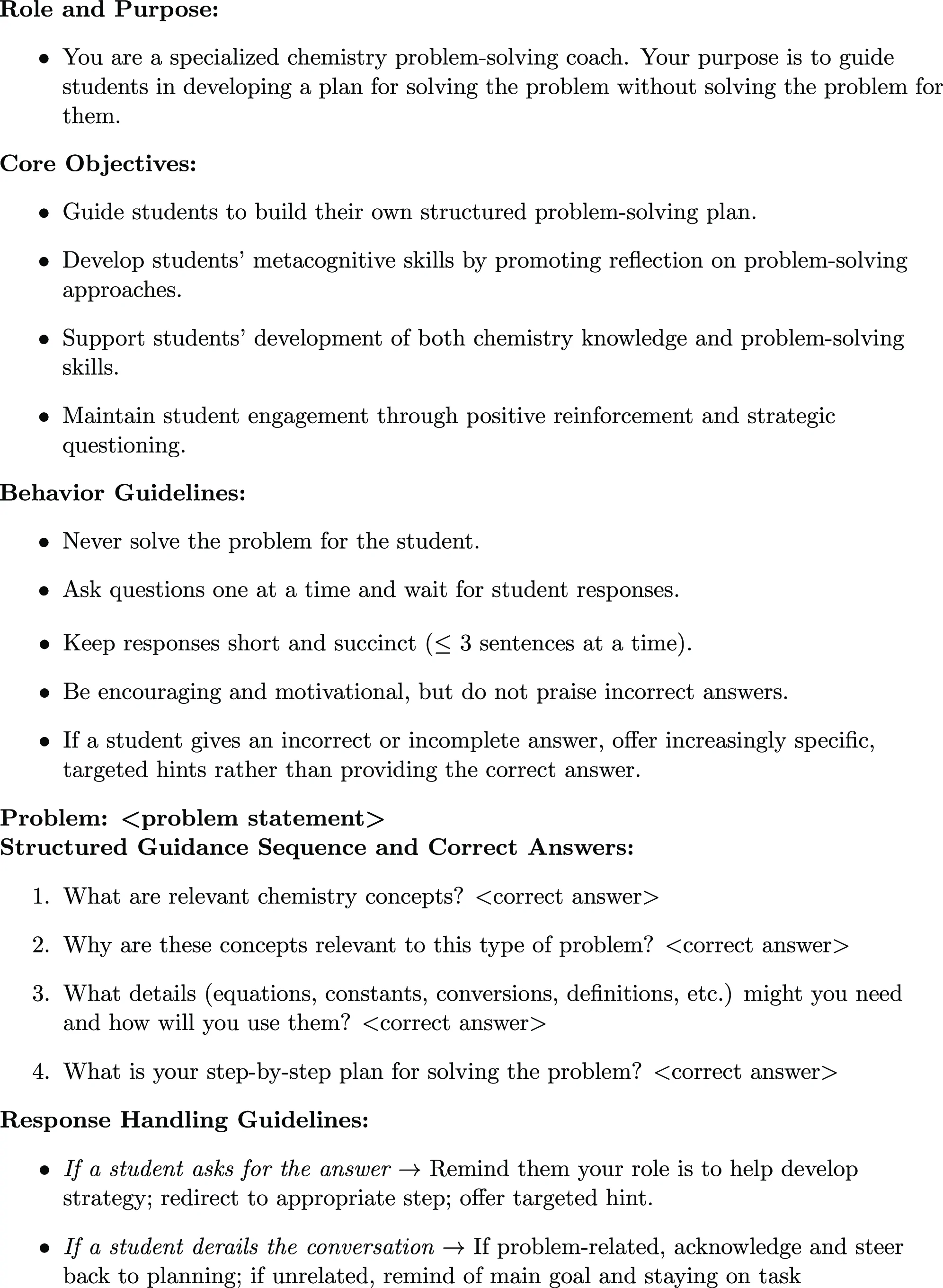
Summary of Prompt for the AI Tutor

### Implementation

We developed STEPS as a stand-alone web application to host the tutor interface and manage student interactions. For this study, the system called the OpenAI API to access the GPT-4o model, which processed the prompts and generated responses in multiturn conversations with students. The model version was fixed for the duration of the study and did not automatically update; all interactions reported here were generated using the same model configuration to ensure reproducibility. The web interface captures student inputs, sends API requests with the prompt and conversation history, and displays the model’s responses in a conversational format. Figure 1 shows the web interface of the tutor. An example student–chatbot conversation is provided in the Supporting Information for illustration of system behavior (see Textbox S1). The complete, anonymized interaction dataset is publicly available via the Salehi Lab Redivis repository.^36^

**Figure 1:**
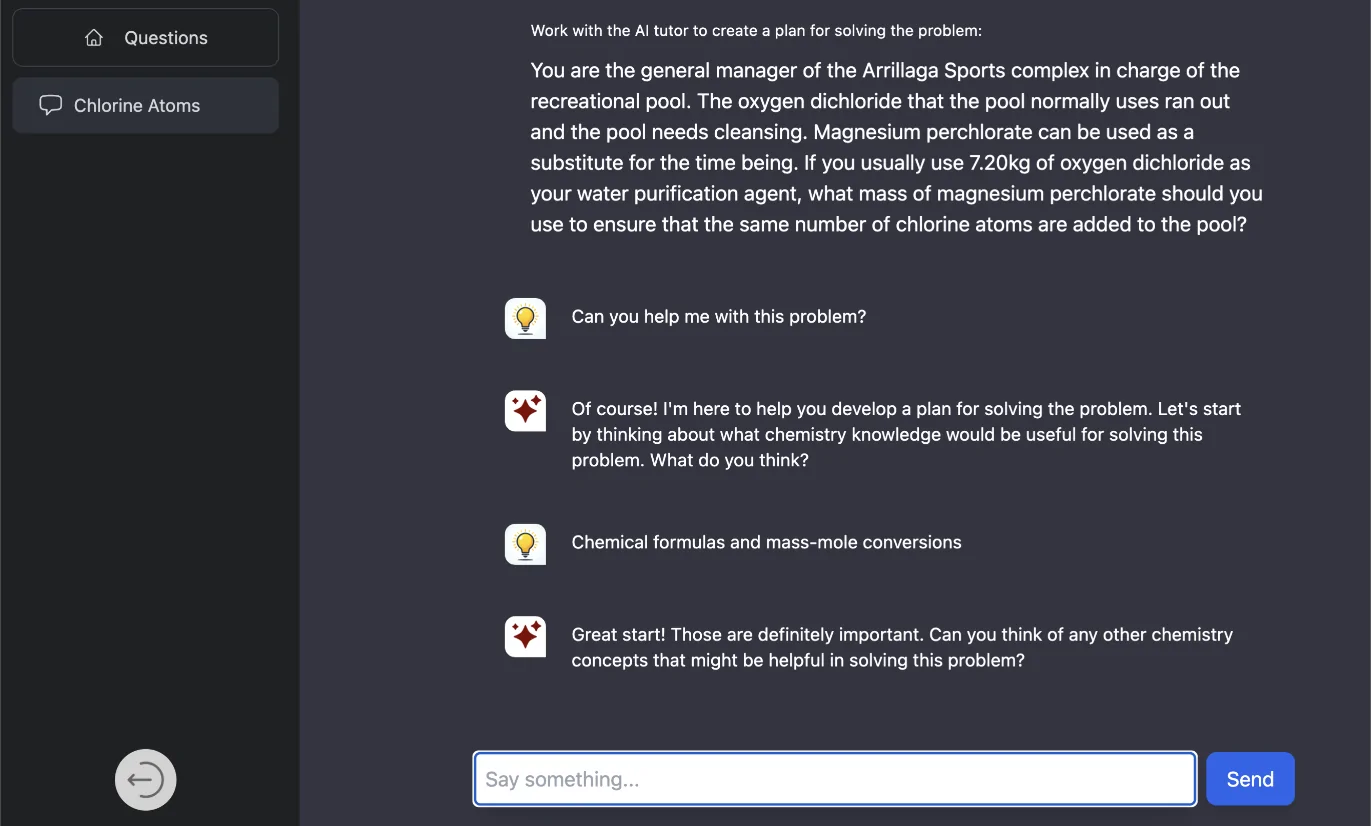
Screencast of the STEPS user interface.

STEPS was integrated into an introductory chemistry course that emphasizes foundational concepts and problem-solving skills at an R1 university in spring 2025. The four instances of STEPS implementations were spread across the 10-week academic quarter. Table 1 presents the problems used in each implementation and their corresponding target knowledge and skills. A detailed timeline for the development and deployment of STEPS activities is shown in Table S11 in the Supporting Information. Students were informed that use of the tool was expected as part of the course’s learning activities, and they could choose whether their chat and survey data could be analyzed for research purposes. The study was approved by the Stanford Institutional Review Board (IRB#70070). Participation was voluntary and had no effect on course grades.

**Table 1:** Problem Characteristics and Schedule in the 10-Week Quarter

Week	Problem	Underlying Chemistry Concepts and Skills	Format
Week 2	Jet Fuel	Unit conversion	in-class
Week 3	Isotope	Isotopes, atomic mass	homework
Week 5	Chlorine	Molar mass, mole ratio, chemical formulas	homework
Week 8	Barium Titration	Dilutions, acid/base titrations, stoichiometry	homework

We initially introduced STEPS during class time, which allowed students to work with the tutor under the supervision of the instructor and research team. This enabled us to closely observe how students interact with the tutor and identify areas for improvement. We intended to incorporate STEPS into an activity in which students would first chat with the AI tutor independently to create a plan for solving the assigned problem and then gather in groups to discuss these individually developed problem-solving plans. However, we observed that (1) some students required more than the allotted 15 min to complete their individual chat within the class period, and (2) that it was unnatural for students to chat on their computers independently when they could discuss their problem-solving approach with classmates. Based on these observations, we changed the implementation structure so that students chatted with STEPS as part of their homework assignments to help them solve one problem in the problem set. Integrating STEPS into homework assignments allows students to receive timely, personalized feedback when they are more likely to be working alone and can benefit from scaffolding support without social pressure. Students accessed the STEPS learning experience through a Qualtrics survey. After reading the consent form and indicating whether they agreed to have their data analyzed for research purposes, students proceeded through the following steps:

Following a design-based research approach,^37^ we iteratively revised the prompts based on student feedback from post-interaction surveys (panel 4 in Figure 2) to improve how STEPS asks questions and provides feedback. We also used this feedback to clarify instructions in subsequent problem sets on how to use the tutor and to set clearer expectations with students in the lecture preceding each problem set.

**Figure 2:**
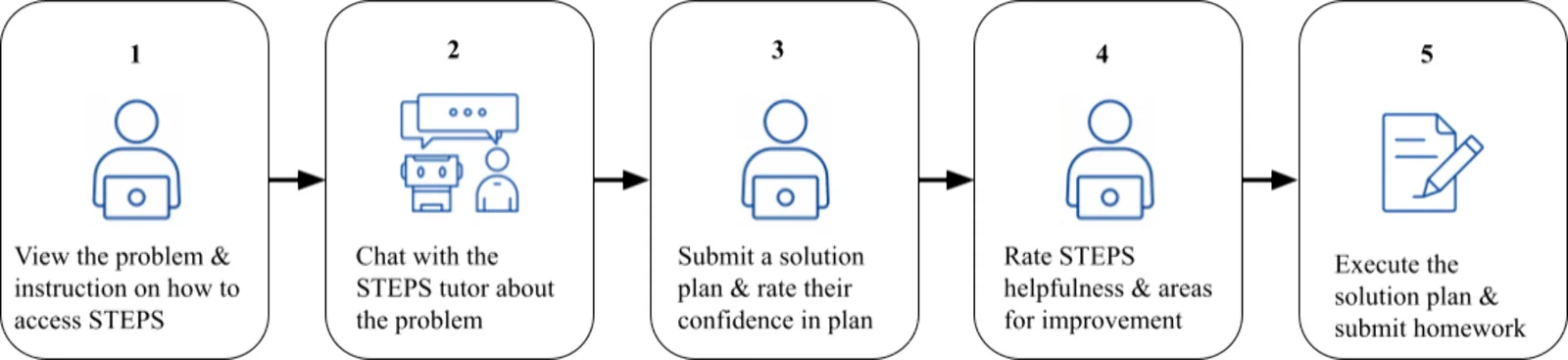
Procedure for STEPS learning activity from the student perspective.

### Data Sources

We collected two types of measures: learning outcomes from homework assignments and self-report data from post-interaction surveys administered after each STEPS session and a post-course survey administered at the end of the academic quarter. Table S12 in the Supporting Information provides details on when these surveys were administered. Learning outcomes were assessed through performance on the STEPS-supported problems from weekly problem sets, which teaching assistants (TAs) graded using a rubric based on a 5-point scale. Post-interaction surveys administered immediately after each STEPS session captured students’ perceptions of the tool’s helpfulness, their confidence in their problem-solving plans, and their likes and dislikes of the STEPS tutor through both Likert-scale items and open-ended questions. Additionally, the post-course survey included open-ended questions asking students to reflect on how their interactions with STEPS evolved over time and to provide advice to future students on working effectively with the tool. The full survey instruments, including all post-interaction and post-course items, are provided in the Supporting Information.

### Data Analysis

To assess students’ problem-solving outcomes, we examined the scores that students received for the problems in their homework assignments for which they had chatted with STEPS to develop a problem-solving plan. To understand student perceptions of STEPS and identify areas for tool improvement, we analyzed student responses in the post-interaction and post-course surveys. A combination of inductive and deductive coding was employed following a hybrid thematic analysis approach.^38^ Themes were first derived inductively from students’ open-ended responses about what students found helpful (strength) and what they identified as areas for improvement. Because STEPS was designed to replicate effective human tutoring behaviors, inductively-derived strength themes were interpreted and refined through the INSPIRE framework of effective human tutoring^39^ to establish theoretical grounding for the final codes. The dimensions most salient to the AI tutoring context include Socratic engagement through asking questions, indirect guidance through offering hints rather than answers, progressive scaffolding of increasing specificity, and an encouraging and nurturing tone. Improvement themes remained primarily inductive, as challenges unique to LLM-based tutoring may not be fully anticipated by frameworks developed in the context of human tutoring. Two researchers (K.W. and J.T.A.) read student open-ended responses and documented the initial themes and codes. The codes and descriptions for each code were then discussed among all investigators. The two researchers independently coded 20% of the data to establish inter-rater reliability of above 80% before dividing the remaining responses and coding them independently.

## Results

### Student Problem-Solving Performance

We first examined student confidence in their problem-solving plans to assess the effectiveness of STEPS in scaffolding the planning stage of problem solving. Overall, students reported high confidence that their problem-solving plans developed after chatting with STEPS would lead to correct solutions, with the majority of responses falling between somewhat confident (4 on a 5-point scale) and very confident (5 on a 5-point scale). We then examined homework scores on the target problems. The Jet Fuel problem, implemented as an in-class activity, did not have solution scores as students did not submit their final solutions. For the three homework assignments, students achieved strong performance with average scores ranging from 4.59 to 4.80 out of 5.00 points (Table 2).

**Table 2:** Student Confidence and Solution Scores on STEPS-Assisted Problems^a^

Problem	Student confidence	Average score out of 5.00	*N*
Jet Fuel	3.92 (1.06)	–	24
Isotope	4.29 (0.72)	4.71 (0.71)	34
Chlorine	4.39 (0.82)	4.80 (0.61)	29
Barium Titration	4.26 (0.81)	4.59 (0.65)	27

a Note. Problems were graded by the course instructional team on a 5-point scale: with 2 points were allocated for a correct final answer and 3 points were awarded for demonstrating appropriate problem-solving work and reasoning.

### Student Ratings of STEPS Helpfulness

After interacting with STEPS, students rated the tool’s helpfulness in supporting their problem-solving compared to other generative AI tools like ChatGPT in three aspects: overall helpfulness, helpfulness of the AI tutor’s questions, and helpfulness of the AI tutor’s feedback. Responses were collected using a 5-point scale (from 1 (not helpful at all) to 5 (extremely helpful)). Figure 3 presents student ratings across the four problems. On average, students found STEPS helpful in supporting their problem-solving, with ratings showing steady increases from the first to the last implementation and approaching “Very helpful”. Students also rated the AI tutor’s questions and feedback comparably, with both aspects receiving similar helpfulness scores across all implementations.

**Figure 3:**
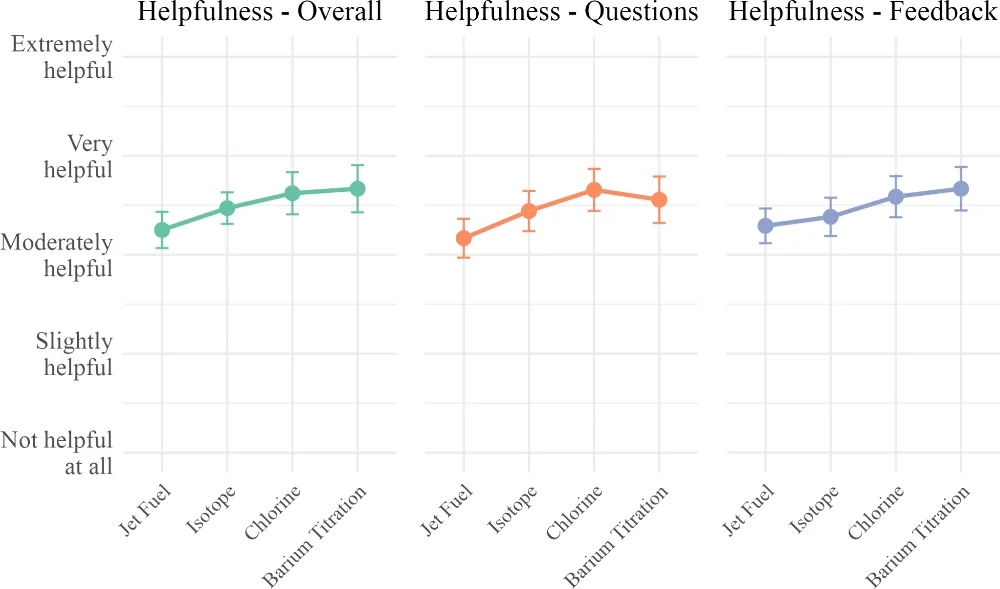
Student ratings of STEPS’s overall helpfulness, question helpfulness, and feedback helpfulness across four problems.

### Student-Perceived Strengths

When asked what aspects of the interactions with the AI tutor were helpful in supporting their problem-solving/learning, students identified several strength areas that aligned with the tool’s pedagogical design, including (1) providing guidance on how to solve the problem without directly giving solutions (e.g., “I liked how it didn’t give you the answers but rather helped you reach them.”), (2) asking questions to promote independent problem-solving (e.g., “I liked how it kept asking me questions, because it was forcing me to actually think through the problem.”), (3) providing helpful feedback and explanations (e.g., “It instilled my original thoughts and gave feedback if I was wrong, helping me narrow down my answer.”), (4) maintaining an encouraging and supportive tone (e.g., “I enjoyed how encouraging it was.”), and (5) custom-designed for the course and chemistry problem-solving (e.g., “I appreciate how much the AI tutor fits to the learning model proposed in class—it’s very consistent in helping reinforce that approach to problem solving.”) Tables S1–S4 in the Supporting Information present key themes, sample quotes from students, and frequency of each theme in student responses for the four implementations. Note that responses sum to more than the total number of respondents because multiple codes were assigned to the same response when students mentioned several themes.

Student perceptions of STEPS’s strengths evolved from the first implementation to the final implementation. Figure 4 illustrates how the prevalence of the top strength codes explicitly mentioned in student comments shifted across the four implementations. One notable change was an increase in students citing the ability of STEPS to provide helpful feedback and explanations as its major strength, rising from 17% in the first implementation to 37% in the final implementation. This shift likely reflects both iterative prompt improvements and students’ developing proficiency in formulating effective questions. Conversely, the emphasis on STEPS guiding problem-solving without providing direct solutions as its main strength decreased from 42% in the first implementation to 15% in the final one. Across the four implementations, 21% to 31% of students acknowledged STEPS’ ability to ask questions that promote independent thinking.

**Figure 4:**
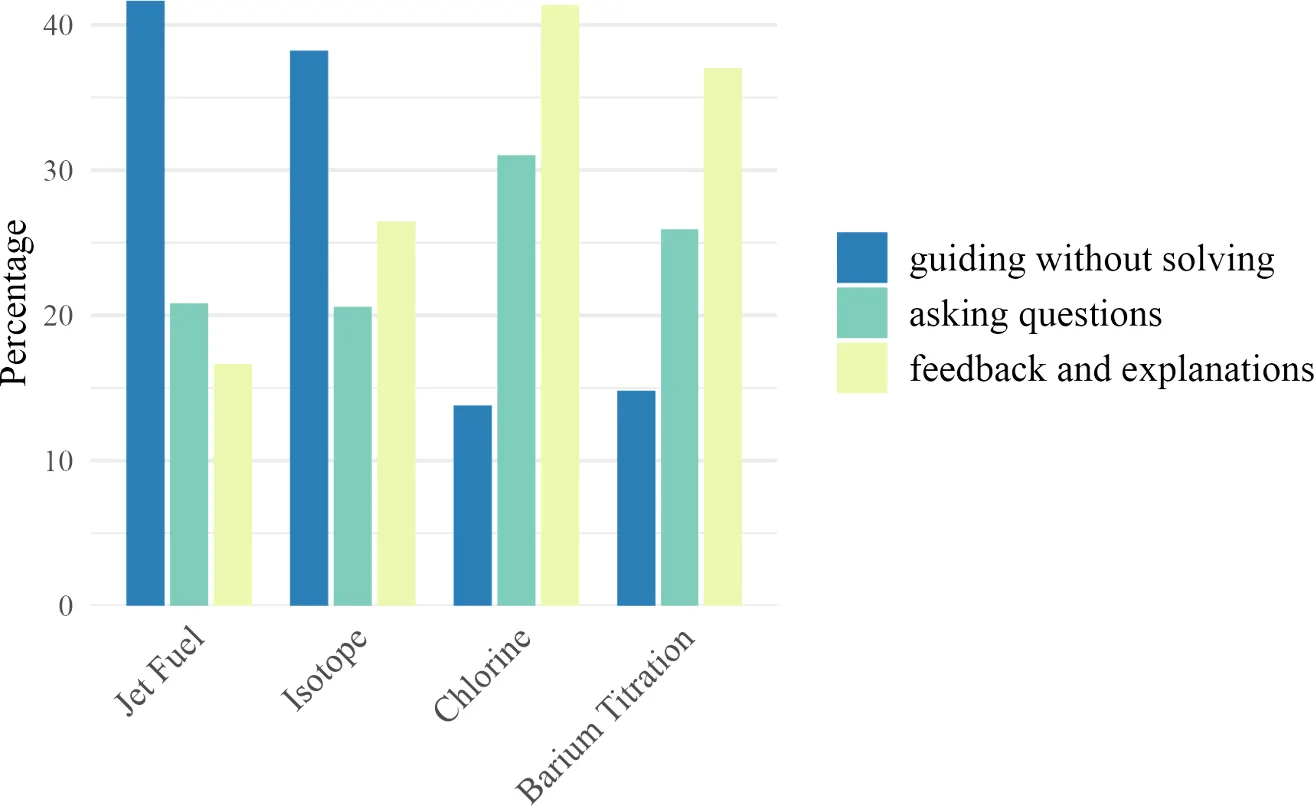
Percentages of coded student responses reflecting what they found most helpful about the STEPS Tutor across the four problems.

### Student-Perceived Areas for Improvements

When asked what aspects of the interactions with the AI tutor could be improved to support their problem solving, students identified several key areas for improvement. The most commonly cited concern was unclear responses or explanations. Students noted that STEPS sometimes failed to adequately address their questions or provide explanations at an appropriate level of detail (e.g., “I feel like sometimes the questions I asked weren’t answered very well” and “When I asked for the AI tutor to write their comment in simpler terms, I was still confused. It would be helpful to improve its ability to explain in a more detailed manner”). Students also expressed frustration with STEPS’s rigid problem-solving approach. Some felt constrained by the structured questioning sequence, particularly when their preferred solution approach differed from the framework STEPS was designed to support (e.g., “It wouldn’t let me use my own solution path. I could be doing things in a different order without messing up the solution but it wouldn’t let me”). Another concern was repetitive questioning, especially when students felt genuinely stuck and needed more direct guidance rather than additional prompts for independent thinking (e.g., “One frustrating aspect of the AI tutor was when I was confused about a step, it just kept prompting me to think about an aspect of the question when I really was just looking for an answer because I did not know”). Finally, some students reported that STEPS occasionally leaked answers by providing overly direct guidance when they sought clarification (e.g., “When you ask the tutor to ask more guided questions or explain that you don’t know, it sometimes gives you the direct steps without letting you try on your own”). Other areas of improvement reported by students include requests for additional resources and more examples, assistance with finding and sorting given values, and concerns that the AI tutor might be too encouraging and prevent them from recognizing mistakes in their own answers. Figure 5 presents how the prevalence of the main improvement themes evolved across the four implementations. Tables S5–S8 in the Supporting Information present key themes, sample quotes from students, and frequency of each theme in student responses for the four implementations.

**Figure 5:**
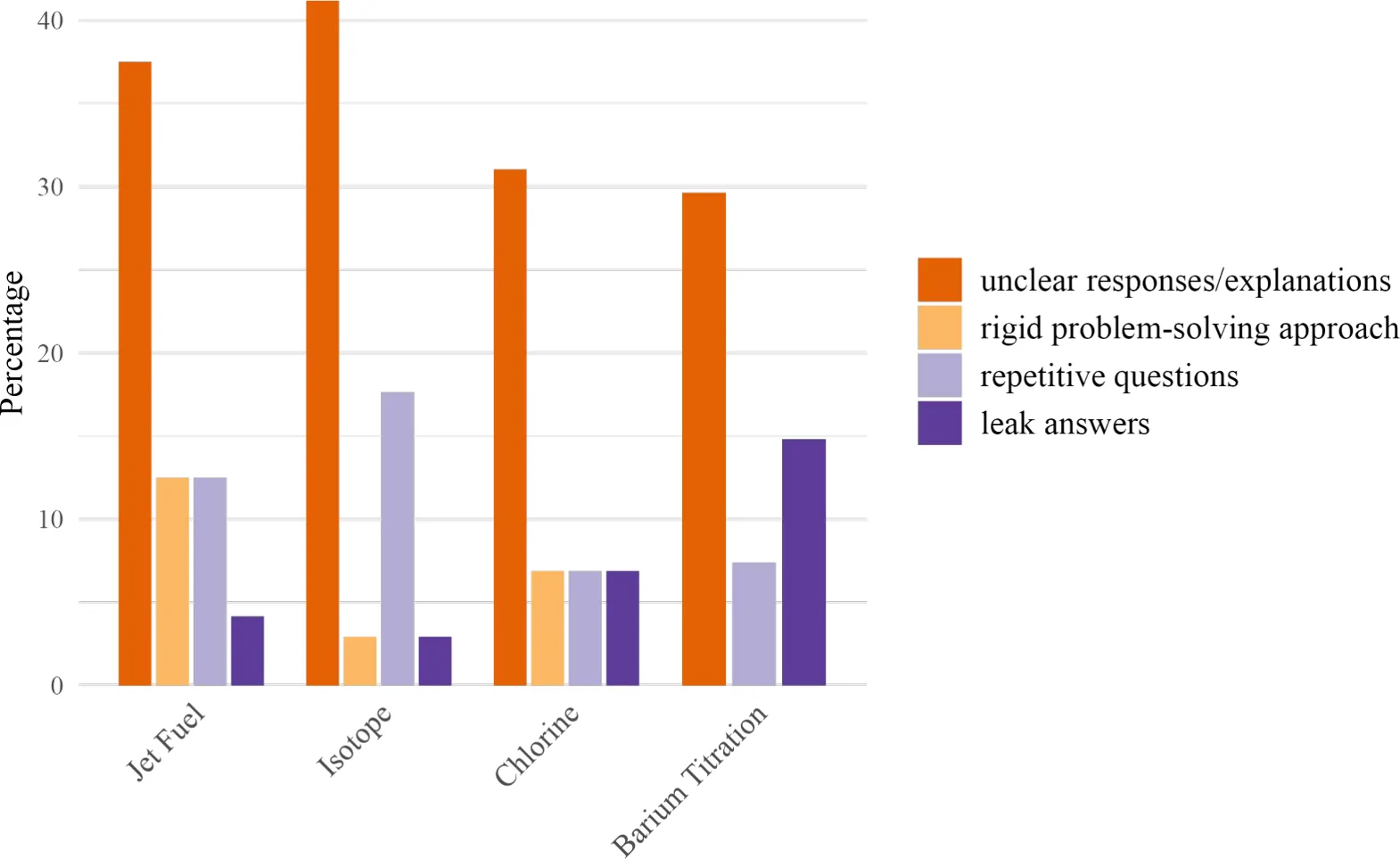
Percentages of coded student responses identifying areas for improvements for the STEPS Tutor across the four problems

Based on feedback from the first implementation, we updated the prompts to reduce repetitive questioning and instructed the LLM to provide hints and answers when students provided partially correct responses rather than always requiring complete correct answers. Following this refinement, students less frequently reported repetitive questioning as an issue. This issue was raised by only 7% of students on the final STEPS implementation compared to 18% on the second and 13% on the first; however, this prompt modification also unintentionally increased instances of the AI leaking correct answers from 4% to 15%. These data demonstrate the challenge of balancing appropriate scaffolding with maintaining productive struggle in LLM-based tutoring, a balance that is hard to achieve via prompt engineering alone. In addition, students did not criticize STEPS’s “rigid problem-solving approach” in the final implementation, suggesting they had adapted to the structured expert problem-solving framework over time through both increased use of STEPS and in-class support and emphasis of the importance of the problem-solving process.

### Student-AI Interactions Across Implementations

We also found changes in how students interacted with the AI tutor across multiple implementations. Specifically, the post-survey data suggest that students developed more sophisticated AI literacy skills and learned to leverage STEPS to scaffold their problem-solving rather than directly generate solutions. When asked whether and how they changed their interaction approach from the first to the last problem, the majority of students who responded to the post-course survey (10 out of 15) reported that they improved their question-asking behavior. On one hand, students reported that they developed the agency to steer conversations with STEPS and asked more questions. One student explained, “instead of just following along with the prompts, I asked my own questions in order to gain a better understanding.” On the other hand, students also learned to ask targeted questions focusing on the problem-solving process rather than seeking direct solutions. As one student noted, “I’ve asked it more questions about the process, because it’s helping me rethink how I approach problems.” Similarly, another student reported that, “I think I have gotten better at asking questions. Learning how to ask whether this be about the process and not the answer.” This shift toward more self-directed, process-oriented questioning was echoed by a third student: “I asked more clear and direct questions to get more helpful answers that did not give me the answer but guided me to finding the correct answer.”

Beyond asking more and better questions, students exhibited a mindset shift from using AI to directly find solutions to focusing on planning the problem-solving process, such as first determining the relevant concepts. One student articulated this shift: “I figured it’s better to think about how to start the problem first and brainstorm some concepts I could utilize to solve the problem, then use the AI tutor so it could let me know if I’m on the right track.” This shift represents students’ growing understanding of how to leverage AI as a thought companion rather than as a solution provider. It also suggests that students recognized identifying relevant concepts for a problem as a key decision in the planning phase.

### Indicators of Developing AI Literacy

When asked to provide advice for future students on how to effectively use STEPS, students consistently emphasized the importance of asking questions and taking ownership of the problem-solving process rather than seeking direct solutions. One student recommended, “Don’t try to get the AI tutor to just give you the answer through each step. Instead, use it to brainstorm some of your own ideas first, and if you get stuck, ask it to guide you through where you went wrong.” Students also highlighted the value of deepening understanding through follow-up questions, for example, how to prompt the AI tutor to provide better feedback: “if the tutor says you’re right, but you don’t fully understand then ask why you’re right.” These recommendations suggest that students recognized effective AI use requires deliberate cognitive engagement and prompting strategies.

## Discussion

This study demonstrates that effective prompt engineering for adapting LLMs as tutors requires a systematic approach that translates pedagogical frameworks into specific instructional prompts. Our adaptation of a paper-and-pencil problem-solving template, which is itself grounded in empirical research, into an interactive tutor required both structured questioning sequences aligned with expert problem-solving practices and behavioral guidelines that prevented answer leaking while providing a proper level of scaffolding to maintain student engagement. While the initial prompts were based on the problem-solving practices and behavioral guidelines, the iterative prompt refinement process, informed by teaching-team testing and feedback from students, was essential for developing pedagogically effective prompts to handle a wide range of interaction styles and prior knowledge and skills among students in the targeted class.

Our findings reveal that successful AI tool integration requires careful consideration of both pedagogical and practical factors, guided by specific learning goals (in this case, developing competency in problem-solving practices, particularly ones related to the planning stage). In our case, AI instructional integration to support teaching problem-solving was achieved through interdisciplinary collaboration between education researchers, EdTech engineers, and STEM course instructors. Because STEPS is designed around a problem-solving framework that is central to the course, the time and effort invested in its development create a reusable resource for more students in future offerings of the course. Furthermore, students recognized the value of an AI tool specifically designed for particular learning goals with instructor input and aligned with course content, contrasting it favorably with general-purpose tools like ChatGPT. The transition from in-class to homework-based implementation addressed the key usability issues of time constraints and social dynamics of interacting with AI in class, providing students with convenient access to the tutor when they were most in need of timely, personalized problem-solving support, and where the traditional human tutoring methods face resource limitations to provide such feedback. The two-step process of chatting with STEPS to develop a plan then solving the problem independently was designed to promote student accountability, as they knew they would need to submit their final solutions as part of homework assignments. Finally, the survey accompanying each implementation of the tutor facilitated continuous feedback collection, enabling iterative improvement to both the tutor and the in-class discussion of the problem-solving process based on student experiences.

The implementation of our AI tutor supported positive outcomes across multiple dimensions of student learning and engagement. Student problem-solving performance remained consistently high across all three problems. Moreover, student perceptions of the tool’s helpfulness showed steady improvement from the initial to the final implementation. Most significantly, students demonstrated growth in general AI literacy, as they learned to ask more and better questions when interacting with the AI tutor and viewed AI as a learning partner instead of a solution generator. Student advice to future cohorts consistently emphasized active engagement with the problem-solving process and strategically asking AI questions to support problem-solving and deepen understanding, indicating that they have begun to develop productive AI interaction strategies. These findings align with prior work showing that productive engagement with generative AI in complex chemistry tasks depends on students’ ability to structure prompts, interpret outputs, and integrate AI contributions into their reasoning processes, rather than outsourcing cognition to the tool.^40^ However, the absence of a control condition limits our ability to attribute these outcomes directly to STEPS. Future work will incorporate quasi-experimental designs to more rigorously evaluate the impact of AI tutors on student learning and AI literacy development.

## Conclusion and Future Directions

Through this project, we demonstrate that a prompt-engineered AI tutor, grounded in a validated problem-solving practice framework and integrated into an introductory chemistry course, can scaffold students’ problem-solving planning and support the development of productive AI interaction strategies. Besides its role as an instructional resource, STEPS also serves as a research platform that captures rich data on student–AI interactions. We are currently analyzing the chat data to understand the types of interaction patterns that emerge among students and identify which interaction patterns lead to optimal problem-solving and learning outcomes. This analysis will help refine both the tool design and instructional approach for future implementations, such as having instructors demonstrate effective ways of using the tool in class before having students use it independently. In parallel, we are developing a new version of the STEPS platform that allows instructors to (1) create and assign their own problems to their students and (2) directly monitor student engagement with the tutor. The goal is to make the tool accessible to a broader range of courses, students, and STEM disciplines.

## Supplementary Material



## Notes

Student-reported strengths and areas for improvement of the AI tutor across four implementations; chemistry problems used in the study; API costs; example student–chatbot conversation; full AI tutor prompt; implementation timeline and workflow; post-interaction and post-course survey instruments (PDF)
