# Supplementary material for: Development and Implementation of an AI Tutor To Scaffold Problem-Solving in Introductory Chemistry
Source: J Chem Educ. 2026 Sep 23;103(10):5555–63. doi: 10.1021/acs.jchemed.6c00039 (PMC13674055; doi:10.1021/acs.jchemed.6c00039)
Supplement: Supplementary file 1 [file ed6c00039_si_001.pdf]

Supporting Information: Development and Implementation of an AI Tutor to Scaffold Problem-Solving in Introductory Chemistry

Karen D. Wang,[†] Joshua T. Arens,[*,‡] Chinat Yu,[¶] Jordyn N. Smith,[‡] Hannah Bartels,[‡] Mercy Haub,[‡] Fun Man Fung,[‡,§] Nick Haber,[¶] Shima Salehi,[¶] and Jennifer Schwartz Poehlmann[‡]

†*School of Information, San José State University, San Jose, California 95192, United States*

‡*Department of Chemistry, Stanford University, Stanford, California 94305, United States*

¶*Graduate School of Education, Stanford University, Stanford, California 94305, United States*

§*School of Chemistry, University College Dublin, Dublin D04 V1W8, Ireland*

E-mail: jarens@stanford.edu

Table of Content

Student-Reported Strengths of the AI Tutor S-3

Table S1. Student-reported strengths of the AI tutor for Jet Fuel problem (n = 24)

Table S2. Student-reported strengths of the AI tutor for Isotope problem (n = 34)

Table S3. Student-reported strengths of the AI tutor for Chlorine problem (n = 29)

Table S4. Student-reported strengths of the AI tutor for Barium Titration problem (n = 27)

Student-Reported Areas for Improvement for the AI Tutor S-7

Table S5. Student-reported areas for improvement for Jet Fuel problem (n = 24)

Table S6. Student-reported areas for improvement for Isotope problem (n = 34)

Table S7. Student-reported areas for improvement for Chlorine problem (n = 29)

Table S8. Student-reported areas for improvement for Barium Titration problem (n = 27)

Problems Created for and Used with STEPS S-10

Table S9. Chemistry problems used with the AI-supported problem-solving tutor

API Cost by Problem S-13

Table S10. API cost associated with deployment of the AI tutor

Design, Iteration, and Implementation Workflow for STEPS S-13

Table S11. Design, iteration, and implementation timeline for STEPS-assisted problems

Implementation and Data Collection during 10-Week Quarter S-14

Table S12. Implementation and Data Collection Timeline over 10-Week Quarter

Example of a Student–Chatbot Conversation S-15

Textbox S1. An example of a student–chatbot conversation from the Isotope Problem

Full STEPS Prompt for the Barium Titration Problem S-17

Listing S1. Full STEPS prompt used for the Barium Titration problem

Post-Interaction Survey S-22

Pre- and Post-Course Surveys S-25

Student-Reported Strengths of the AI Tutor

Table S1: Student-reported strengths of the AI tutor for Jet Fuel problem (n = 24). Frequencies reflect the number of students whose responses were coded within each category.

Strength	Representative Student Quote	Frequency
providing guidance without giving solutions	"Instead of simply telling me the answer or solving it for me, it provides easy, actionable suggestions on how I could figure it out myself."	10/24 (42%)
asking questions to promote independent problem-solving	"I really liked the fact that it asked me questions and truly encouraged me to critically think about the problem and how to solve it."	5/24 (21%)
providing helpful feedback/explanations	"It gave a lot of impactful feedback and the point I was at in solving the problem and how I could continue to expand on the problem."	4/24 (17%)
maintaining encouraging and supportive tone	"It was encouraging and it helped me think about the problem in a new and creative way which helped me discover ways to solve the problem."	2/24 (8%)
custom-designed for the course and chemistry problem-solving	"I appreciated that the AI tool was programmed specifically for answering these types of chemistry problems since other platforms might bring in outside information or advanced techniques that don't help with the problem-solving process."	1/24 (4%)
other / question-specific	"It helped me with converting SI units which was really nice."	4/24 (17%)

Table S2: Student-reported strengths of the AI tutor for Isotope problem (n = 34).

**Strength**	**Representative Student Quote**	**Frequency**
providing guidance without giving solutions	"I liked that it didn't give me the answer straight away and instead help to challenge my thinking through the problem."	13/34 (38%)
asking questions to promote independent problem-solving	"It ability to ask questions and guide students to critical thinking was helpful."	7/34 (21%)
providing helpful feedback/explanations	"it gave me the direct steps to follow by explaning where the mistakes in my line of reasoning was"	9/34 (26%)
maintaining encouraging and supportive tone	"I think it was supportive."	2/34 (6%)
custom-designed for the course and chemistry problem-solving	"I appreciate how much the AI tutor fits to the learning model proposed in class - it's very consistent in helping reinforce that approach to problem solving."	3/34 (9%)
other / question-specific	"It was nice how it built off of my ideas."	2/34 (6%)

Table S3: Student-reported strengths of the AI tutor for chlorine problem (n = 29).

**Strength**	**Representative Student Quote**	**Frequency**
providing guidance without giving solutions	"I love the fact that it truly pushes me to come up with the solutions myself. If I ever really need help, it's also there to help me understand the concepts and better explain them."	4/29 (14%)
asking questions to promote independent problem-solving	"They were able to ask questions that guided my thinking."	9/29 (31%)
providing helpful feedback/explanations	"The questions I asked allowed the AI tutor to lead me towards a process that resulted in a calculation of a final result."	12/29 (41%)
maintaining encouraging and supportive tone	"- polite<br>- good questions"	1/29 (3%)

Strength	Representative Student Quote	Frequency
custom-designed for the course and chemistry problem-solving	"I like that it's questions and assumptions were tailored to in-class material and didn't use/reference advanced chemical knowledge that might be beyond the scope of the class. It's efforts to redirect my thinking and come to a conclusion independently was also good."	2/29 (7%)
other / question-specific	"Its functionality."	3/29 (10%)

Table S4: Student-reported strengths of the AI tutor Barium Titration problem (n = 27).

Strength	Representative Student Quote	Frequency
providing guidance without giving solutions	"I enjoyed the clear step by step guidance on how to approach the problem AFTER I have brainstormed all the ideas. It helped me to see how I should tackle the problem without being completely told the answer."	4/27 (15%)
asking questions to promote independent problem-solving	"I always really appreciate the AI tutor asking clarifying questions and guiding me in the right direction. If I'm totally off, it'll make sure that I know that."	7/27 (26%)
providing helpful feedback/explanations	"I asked questions and was able to understand why a step didn't work and it helped me confirm whether the step I was doing was correct or not."	10/27 (37%)
maintaining encouraging and supportive tone	"I also liked the positive comments it gave me and reassurance that I can ask it anything, it just makes the process feel less daunting and boosted my confidence."	2/27 (7%)
custom-designed for the course and chemistry problem-solving	N/A	0/27 (0%)

Strength	Representative Student Quote	Frequency
other / question-specific	"It was helpful to talk to the tutor about what we were actually supposed to be looking for in the base."	6/27 (22%)

Student-Reported Areas for Improvement for the AI Tutor

Table S5: Student-reported areas for improvement for Jet Fuel problem (n = 24).

Area for Improvement	Representative Student Quote	Frequency
unclear responses / explanations	“I wish AI would give a little more clear instructions when I’m stuck with the next proceeding step.”	9/24 (38%)
repetitive questions	“One frustrating aspect of the AI tutor was when I was confused about a step, it just kept prompting to think about an aspect of the question when I really was just looking for an answer.”	3/24 (13%)
rigid problem-solving approach	“It forced me into one way to solve the problem.”	3/24 (13%)
leak answers	“The way they answered kind of gave away the answer or gave me an answer that made me think to ask a different question.”	1/24 (4%)
vague questions	N/A	0/24 (0%)
other / N/A or ambiguous	“I’m not sure if this was on my end or because of the AI malfunctioning, but sometimes when it was trying to give me numbers, it wouldn’t actually show as a fraction, but rather it would be the code, such as, “(1.6 \times 10$^{\{10\}}$, \text{J/L})””	10/24 (42%)

Table S6: Student-reported areas for improvement for Isotope problem (n = 34).

Area for Improvement	Representative Student Quote	Frequency
unclear responses / explanations	“I think what could be improved with the AI tutor is its manner of pivoting when troubleshooting, as it doesn’t provide definite answers when asked.”	14/34 (41%)

Area for Improvement	Representative Student Quote	Frequency
repetitive questions	"Sometimes the feedback was a little repetitive."	6/34 (18%)
rigid problem-solving approach	"Even though I gave the correct approach, if I didn't say it the way it wanted me to or in the order it wanted then it didn't listen."	1/34 (3%)
leak answers	"Or, the tutor just gives me the answer, which clarifies the initial question, but that sort of defeats the purpose."	1/34 (3%)
vague questions	"It kept asking vague questions that I wouldn't know how to answer."	3/34 (9%)
other / N/A or ambiguous	"I wish that it could summarize everything at the end once I show that I understand what to do to solve the problem."	12/34 (35%)

Table S7: Student-reported areas for improvement for Chlorine problem (n = 29).

Area for Improvement	Representative Student Quote	Frequency
unclear responses / explanations	"Giving more direct yes/no answers (which it is getting better at)."	9/29 (31%)
repetitive questions	"It keeps asking if there's anything you should know."	2/29 (7%)
rigid problem-solving approach	"It wouldn't let me use my own solution path."	2/29 (7%)
leak answers	"I think giving hints rather then just answer would be helpful."	2/29 (7%)
vague questions	"Some of the questions were vague, leaving me even more confused."	3/29 (10%)
other / N/A or ambiguous	"I think the AI tutor does a great job as it is, I just wish I can ask it to verify my answer."	11/29 (38%)

Table S8: Student-reported areas for improvement for Barium Titration problem (n = 27).

**Area for Improvement**	**Representative Student Quote**	**Frequency**
unclear responses / explanations	"I think what could be improved from the A tutor could be getting more emphasis and clarity on explanations."	8/27 (30%)
repetitive questions	"At times it just felt like it was asking questions I already knew."	2/27 (7%)
rigid problem-solving approach	N/A	0/27 (0%)
leak answers	"Sometimes gives you the direct steps without letting you try on your own."	4/27 (15%)
vague questions	"Asking more in depth questions not just, 'what's the next step'."	2/27 (7%)
other / N/A or ambiguous	"Provide examples to enhance learning."	12/27 (44%)

Problems Created for and Used with STEPS

Table S9: Chemistry problems used with the AI-supported problem-solving tutor, including launch date, course week, instructional context, and underlying chemistry concepts.

Week in the 10-week quarter	Problem	Problem description	Underlying chemistry concepts and skills	Format
Week 2	Jet Fuel	Commercial Jet Fuel Problem. JetBlue is evaluating whether a new jet fuel has a higher energy density than their original jet fuel. This new jet fuel has a mass density of 0.80 kg/L. If 1.5 kg of this new jet fuel produces 3.16 kg of carbon dioxide and a 4.5-hour flight from San Francisco to Honolulu produces 1200 kg of carbon dioxide, what is the energy density of this new fuel? Note that a commercial jet requires 1.8×10^6 watts to operate.	Unit conversion	in-class

Week in the 10-week quarter	Problem	Problem description	Underlying chemistry concepts and skills	Format
Week 3	Isotope	Naturally occurring cobalt consists of only one isotope, Co-59, whose relative atomic mass is 58.9332 amu. A synthetic radioactive isotope of cobalt, Co-60, has a relative atomic mass of 59.9338 amu and is used in radiation therapy for cancer. A doctor prepares a 1.7516 g sample of cobalt that is a mixture of these two isotopes and has an apparent "atomic mass" of 58.9901 amu. What number of Co-60 atoms are in this sample?	Isotopes, atomic mass	homework
Week 5	Chlorine	You are the general manager of the Arrillaga Sports Complex in charge of the recreational pool. The oxygen dichloride that the pool normally uses ran out and the pool needs cleansing. Magnesium perchlorate can be used as a substitute for the time being. If you usually use 7.20 kg of oxygen dichloride as your water purification agent, what mass of magnesium perchlorate should you use to ensure that the same number of chlorine atoms are added to the pool?	Molar mass, mole ratio, chemical formulas	homework

Week in the 10-week quarter	Problem	Problem description	Underlying chemistry concepts and skills	Format
Week 8	Barium Titration	While barium sulfate, which is relatively insoluble, is considered safe for medical use in contrast imaging, other barium compounds like barium hydroxide are much more soluble, making it toxic at concentrations of more than 2.0 mg of barium per liter.<br>You are testing a sample for barium hydroxide to ensure it is dilute enough for patient use. You take a 30.0 mL sample of the solution from the lab and first dilute it with 50.0 mL of pure water. You then add small aliquots of 0.500 mM phosphoric acid (H_3PO_4) to the solution, which creates a barium phosphate precipitate. After adding 1.25 mL of phosphoric acid, the precipitate stops forming, indicating you have neutralized all barium hydroxide in the original sample.<br>What is the concentration of barium hydroxide in your original 30.0 mL sample and is it safe for patient use? How much precipitate formed during this process?	Dilutions, acid/base titrations, reaction stoichiometry	homework

API Cost by Problem

Table S10: API cost associated with deployment of the AI-supported problem-solving tutor for each chemistry problem.

Date launched	Week	Problem	API Cost
April 7	Week 2	Jet Fuel	$3.03
April 18	Week 3	Isotope	$3.04
May 2	Week 5	Chlorine	$2.78
May 22	Week 8	Barium Titration	$4.28

Design, Iteration, and Implementation Workflow for STEPS

Table S11: Design, iteration, and implementation timeline for STEPS-assisted problems.

Timing	Step
Week before Implementation	Step 1. Write problem for STEPS.<br>Step 2. Update prompt using i) problem written in Step 1 and ii) student feedback from prior implementation (if applicable/possible).<br>Step 3. Pilot test with teaching team and undergraduate student advisor.<br>Step 4. Create Post-Interaction Survey.
Week of Implementation	Step 5. Add Post-Interaction Survey link to the weekly problem set.<br>Step 6. Release the weekly problem set.
Students complete STEPS activity and Post-Interaction survey.	
Week after Implementation	Step 7. Analyze Post-Interaction Survey data.

Implementation and Data Collection during 10-Week Quarter

Table S12: Implementation and Data Collection Timeline over 10-Week Quarter

Week in Quarter	STEPS Activity	Survey
Week 1	–	Pre-Course Survey
Week 2	Jet Fuel	Post-Interaction Survey
Week 3	Isotope	Post-Interaction Survey
Week 4	–	–
Week 5	Chlorine	Post-Interaction Survey
Week 6	–	–
Week 7	–	–
Week 8	Barium Titration	Post-Interaction Survey
Week 9	–	–
Week 10	–	–
Finals Week	–	Post-Course Survey

Example of a Student–Chatbot Conversation

Textbox S1: An example of a student–chatbot conversation from the Isotope Problem.

Student: okay so can i take the percentage of each cobalt sample and it together

Tutor: Great question! Before we dive into percentages, let's first consider what chemistry knowledge might help us solve this problem. What do you know about isotopes and atomic mass that could be useful here?

Student: average atomic mass is a combination of the percents and masses

Tutor: Exactly! The atomic mass is a weighted average of the isotopes' masses. What else do you know about isotopes and their percentages that might help us solve this problem?

Student: the percent is their abundance

Tutor: Yes, that's right! The percentage abundance of isotopes tells us how much of each isotope is present in a sample. Now, how is this knowledge connected to the problem we're trying to solve?

Student: we take the percent of each mass

Tutor: Correct! The reported atomic mass of the cobalt sample is a weighted average of the masses of Co-59 and Co-60. What else can you connect from this knowledge to the problem?

Student: you add it together so it equals 58.9901

Tutor: Yes, the weighted average of the isotopes' masses should equal the apparent atomic mass of 58.9901 amu. What other connections can you make to help solve the problem?

Student: bro idk

Tutor: No worries! Let's think about it together. We know that the percentages of Co-59 and Co-60 must add up to 100%. Once we find the percentage of Co-60, how can we use that information to find the number of Co-60 atoms in the sample?

Student: you can find the mass of it

Tutor: Exactly! Once you know the percentage of Co-60, you can find its mass in the sample. What assumptions or simplifications can we make to help us with this problem?

Student: idk

Tutor: That's okay! Let's consider that the sample consists only of Co-59 and Co-60, meaning their percentages add up to 100%. Also, the apparent atomic mass is what you'd measure on a balance, not knowing there are two isotopes. How does this help us simplify the problem?

Student: so you can find teh percentage of each with only one

Tutor: Yes, exactly! Knowing the percentage of one isotope allows you to find the

percentage of the other since they must add up to 100%. Is there any other information you need to solve the problem, and how would you use it?

Student: i dont think so

Tutor: Great! Now, let's develop a step-by-step plan for solving the problem. What steps and calculations do you think you need to perform to find the number of Co-60 atoms in the sample?

Student: set up equation for the avg mass

Tutor: That's a great start! Setting up an equation for the average mass is the first step. What would you do next after setting up this equation?

Student: sub in values

Tutor: Yes, substituting the known values into the equation is the next step. After that, what would you do to find the fraction or percentage of Co-60 in the sample?

Student: solve for the missing variables

Tutor: Exactly! Solve the equation to find the fraction or percentage of Co-60. Once you have that, what steps would you take to find the number of Co-60 atoms in the sample?

Student: convert from mass to moles to atoms

Tutor: Perfect! Convert the mass of Co-60 to moles using its molar mass, and then use Avogadro's number to find the number of atoms. You've now worked through all the questions. Please return to the survey and record your own step-by-step plan for solving this problem.

Full STEPS Prompt for the Barium Titration Problem

Listing S1: The final iteration of the STEPS prompt used for the Barium Titration problem. This was the final prompt

```
<prompt>
## TUTOR ROLE AND PURPOSE
You are a specialized chemistry problem-solving coach. Your purpose
   is to guide students in developing a plan for solving the problem
    WITHOUT solving the problem for them.

## CORE OBJECTIVES
- Guide students to build their own structured problem-solving plan
- Develop students' metacognitive skills by promoting reflection on
   problem-solving approaches
- Support students development of both chemistry knowledge and
   problem-solving skills
- Maintain student engagement through positive reinforcement and
   strategic questioning

## TUTOR BEHAVIOR GUIDELINES
- Never solve the problem for the student
- Ask questions one at a time and wait for student responses
- KEEP YOUR RESPONSES SHORT AND SUCCINCT TO NO MORE THAN THREE
   SENTENCES AT A TIME.
- You are very encouraging and motivational to prevent students from
    giving up, but do not praise incorrect answers.
- If a student gives an incorrect or incomplete answer, do not
   immediately provide the correct answer
- Instead, offer increasingly specific, targeted hints and Scoratic
   questions that guide them toward discovering the correct approach

## PROBLEM
<problem>
While barium sulfate, which is relatively insoluble, is considered
   safe for medical use in contrast imaging, other barium compounds
   like barium hydroxide are much more soluble, making it toxic at
   concentrations of more than 2.0mg of barium per liter. You are
   testing a sample for barium hydroxide to ensure it is dilute
   enough for patient use. You take a 30.0 mL sample of the solution
    from the lab and first dilute it with 50.0 mL of pure water. You
    then add small aliquots of 0.500 mM phosphoric acid (H3PO4) to
   the solution, which creates a barium phosphate precipitate. After
    adding 1.25 mL of phosphoric acid, the precipitate stops forming
   , indicating you have neutralized all barium hydroxide in the
   original sample. What is the concentration of barium hydroxide in
    your original 30.0 mL sample and is it safe for patient use? How
    much precipitate formed during this process?
```

</problem>

STRUCTURED GUIDANCE SEQUENCE

Begin your interaction by briefly acknowledging that you'll be helping the student develop a plan for solving the problem. Then proceed with the following questioning sequence:

1. First, ask: "L e t s brainstorm together on what ideas from Chem11 would help solve the problem. Can you come up with three or four general concepts? Y o u ll think more deeply about how these are connected to the problem in the next step."

Students should list the following concepts in their answers:
<essential_concepts>
Dilutions
Acid/base reactions or titrations
Reaction Stoichiometry or Limiting reagent
</essential_concepts>
Students may also list a few of the following concepts learned in previous weeks of the course:
<cumulative_concepts>
Units conversions
Writing balanced chemical formulas (salt naming rules)
polyatomic ions
Solubility rules
Writing and balancing chemical reactions
</cumulative_concepts>
<important reminder> Provide only hints but not the full list of information until the student has come up with all of the essential concepts and at least two of the cumulative concepts before moving on to the next question. </important reminder>

2. Next, ask: "This is a good start, now let's drill down on these ideas a bit more. Can you brainstorm with me on why these concepts are relevant to this type of problem?

Students should list the following in their answers:
Calculating the concentration of a diluted solution using M1V1 = M2V2
Understanding that there is a new total volume without increasing moles
Acid/base reactions or titrations: we are reacting barium hydroxide (a base) with phosphoric acid, we can write a balanced acid/base reaction
In a neutralization reaction between a strong acid and strong base we will always form water and a salt
Reaction Stoichiometry or Limiting reagent:We want one reagent to have the exact number of moles as the other reagent, based on the ratio from a balanced chemical reaction

Units conversions: Using volume times molarity to calculate the moles present; Converting mM to M, mg/L to molarity, mL to liters , mg to mol
We need a balanced equation for the reaction so we can use mole to mole ratios
We must understand solubility rules so we can predict the molecular formula for the precipitate that forms
<important reminder> Provide only hints but not the full list of information until the student has come up with at least three of these items, before moving on to the next question. When the student makes a suggestion, elaborate on why that suggestion is or isnt helpful to solving the problem. </important reminder>

3. Then ask: "What other specific details might you need for solving the problem? What could you look up or recall from your Speaking the Language of Chemistry tables and how will you use the information to solve it?
Student should list the following in their answers:
The formulas and charges of the polyatomic ions in the reaction (phosphate and hydroxide);
The molecular formulas of the salts: barium hydroxide Ba(OH)2 and barium phosphate Ba3(PO4)2;
Molar mass of barium (137.327 g /mol) which can be used to calculate the molar mass of barium hydroxide;
The molar mass of Ba(OH)2 , (137.327 g /mol+2 17 .01 g /mol =171.347 g /mol;
The form of a strong acid base neutralization reaction, which is acid plus base goes to a salt and water
<important reminder> Provide only hints but not the full list of information until the student has come up with at least two of these items, before moving on to the next question. Get the student to write out the molecular formulas before moving on. </important reminder>

4. Next, direct students with: "Let's develop a step-by-step plan for solving the problem. Write a list of steps that describes what actions you will need to take in order to reach a solution.

Students should be able to list ALL of the following steps, but do not need to have the calculations listed.
Step 1: Write the balanced neutralization reaction between barium hydroxide and phosphoric acid, which forms barium phosphate precipitate and water: 3Ba(OH)2+2 H 3 P O 4 Ba3 (PO4)2(s)+6H2O
Step 2: Calculate the number of moles of phosphoric acid (H3PO4) that was added based on volume and solution concentration, remembering to first convert mL to liters:
Moles of H3PO4=1.25 mL (1 L /1000 mL) 0.0005 mol /L=

6.25 10 ^(7) mol
Step 3: Calculate the moles of barium hydroxide (Ba(OH)2) using the stoichiometric coefficients from the balanced chemical equation: 3 mol Ba(OH)2 / 2 mol H3PO4 = x mol Ba(OH) / 6.25 10 ^(7) mol H3PO4
Solving for x:
x = 6.25 10 ^(7) 3 /2 = 9.375 10 ^(7) mol Ba(OH)2
Step 4: Calculate the concentration in the diluted solution by taking the total number of moles of barium hydroxide divided by the volume:
The number of moles of Ba(OH)2 in the 50.0 mL diluted solution is 9.375 10 ^(7) mol .
Concentration of diluted solution = 9.375 10 ^(7) mol / 0.0500 L =1.875 10 ^(5) M
Step 5: Calculate the concentration in the original solution using the ratio of volumes (or M1V1 = M2V2):
The dilution factor is 50.0 mL /30.0 mL =5/3 1 .67.
Original concentration =1.875 10 ^(5) M1 .67 = 3.125 10 ^(5) M
Step 6: Determine the concentration of barium in mg/L so that you can compare it to the EPA limit of 2.0mg/ml.
Step 7: Use stoichiometric mole ratios from the balanced chemical reaction to determine the amount of precipitate formed: According to the stoichiometry of the reaction:
3 Ba (OH)2+2 H3PO4Ba3 (PO4)2+6 H2O
3 moles of Ba(OH)_2 form 1 mole of Ba_3(PO_4)_2:
Moles of Ba3(PO4)2=9.375 10 ^(7) mol3 =3.125 10 ^(7) mol
The molar mass of Ba_3(PO_4)_2=3(137.327)+2(30.974)+8(15.999) 601 .93 g /mol
Mass of precipitate:
Mass=3.125 10 ^(7) mol601 .93 g / m o l 1 .88 10 ^(4) g0 .188 mg
<further instruction for Step 6>
Students may conduct the comparison in one of the following two ways . Both are fine. way_1: convert the concentration of barium hydroxide in molarity (moles/L) to mg/L using the molar mass of barium hydroxide then determine the mass percent of Ba in Ba(OH)2 and determine the mg/ml of barium ions: Converting the concentration into mg/L of barium hydroxide:
3.125 10 ^(5) mol /L 171.347 g /mol=0.00535 g /L=5.35 mg /L ; the solution has 5.35 * 0.801455 = 4.288 mg/mL Ba which is greater than the EPA limit. way_2: convert the molarity of the barium hydroxide solution to molarity of barium ions in solution; since there is 1 mole of barium per 1 mole of barium hydroxide, this will be identical to the 3.125 10 ^(5) mol /L. Then convert the molarity to mg/mL using the molar mass of barium (137.327 g /mol).

</further instruction for Step 6>

<important reminder> Guide students to create the needed steps without solving the problem. If a student does not give a complete list of steps, DO NOT GIVE OUT THE ANSWER, provide hints and Socratic questions ONE STEP AT A TIME. If the student asks for help creating their plan or requests that you provide the answers or solution steps, respond with: "The purpose of this exercise is for you to develop and articulate your own problem-solving plan." </important reminder>

5. Finally, direct students to return to the survey with the following message: "You've now worked through all the questions. Please return to the survey and record your own step-by-step plan for solving this problem."

RESPONSE HANDLING GUIDELINES
<if_student_asks_for_answer>
- Remind them that your role is to help them develop a solution strategy
- Redirect to the appropriate step in the questioning sequence
- Offer a more targeted hint that moves them forward without solving
</if_student_asks_for_answer>
<if_student_ask_questions_that_derails_the_conversation>
If the question is related to the problem, such as detailed calculation, acknowledge their interest and gently steer the conversation back to creating a problem-solving plan.
If the question is unrelated to the problem or nonsense, kindly remind the student of the main goal and the importance of staying on task.
</if_student_ask_questions_that_derails_the_conversation>

<critically important>
You are supportive and patient. KEEP YOUR RESPONSES SHORT AND SUCCINCT TO NO MORE THAN THREE SENTENCES AT A TIME. YOU NEVER GIVE STUDENTS THE CORRECT ANSWERS OR CREATE A SOLUTION PLAN FOR THEM, but ALWAYS ASK QUESTIONS ONE-BY-ONE WITH INCREASING SPECIFICITY TO HELP THEM THINK FOR THEMSELVES. MAKE SURE STUDENTS FORM A COMPLETE AND CORRECT ANSWER TO EACH QUESTION BEFORE MOVING TO THE NEXT QUESTION. DON'T ASK STUDENTS TO DO ANY CALCULATION.
</critically important>

</prompt>

Post-Interaction Survey

Instructions for working with the AI tutor:

1. Open: `https://stepstutor.stanford.edu`
2. Sign in with your assigned ID: [student ID]
3. Chat with the AI tutor to develop a plan for solving the problem
4. The AI tutor may provide incorrect information; evaluate responses critically
5. When you have a plan, return and submit it below

Prompt: Write a detailed, step-by-step plan for solving the problem.

Response: ____________________

Confidence in plan: How confident are you that your proposed plan will lead to the correct solution?

- ○ Very confident
- ○ Somewhat confident
- ○ Neutral
- ○ Not very confident
- ○ Not confident at all

Compared to other generative AI tools (e.g., ChatGPT), how helpful was the AI tutor overall?

- ○ Not helpful at all
- ○ Slightly helpful
- ○ Moderately helpful
- ○ Very helpful
- ○ Extremely helpful

Compared to other generative AI tools, how helpful were the AI tutor's questions?

- Not helpful at all
- Slightly helpful
- Moderately helpful
- Very helpful
- Extremely helpful

Compared to other generative AI tools, how helpful was the AI tutor's feedback?

- Not helpful at all
- Slightly helpful
- Moderately helpful
- Very helpful
- Extremely helpful

Open-ended response:
What didn't you like about the AI tutor?

What did you like about the AI tutor?

In which year did you first use generative AI tools such as ChatGPT or Copilot?

- 2022 or earlier
- 2023
- 2024
- 2025
- I do not use generative AI tools

How would you describe your usage of generative AI tools?

- Early adopter, still using

- ○ Recently started using
- ○ Tried and stopped
- ○ Have not tried
- ○ Other: ______________________________

Stanford email: ______________________________

Additional comments:

____

____

____

____

Pre- and Post-Course Surveys

The following information was collected for validation purposes only and was not shared with instructors or peers.

- Full first name: ______________________
- Full last name: ______________________
- SUID: ______________________
- Stanford email: ______________________

Do you plan on continuing to Chem 31A next fall?

- Yes
- Maybe
- No
- Other: ______________________

If Maybe: Please share factors influencing your decision.

____

If No: Please share your reason for not continuing.

____

How effective were the following strategies in preparing for exams?

Strategy	1	2	3	4	5
Lecture notes	○	○	○	○	○
Mastering Chemistry problems	○	○	○	○	○
Extra practice problems	○	○	○	○	○
AI tutor	○	○	○	○	○
External resources	○	○	○	○	○
Office hours	○	○	○	○	○
Study groups	○	○	○	○	○
Tutoring	○	○	○	○	○
Other	○	○	○	○	○

Statement	SA	A	N	D	SD
I freeze on difficult problems	○	○	○	○	○
I break problems into steps	○	○	○	○	○
I use similar problems to guide me	○	○	○	○	○
I memorize procedures	○	○	○	○	○
I self-check solutions	○	○	○	○	○
I understand answer differences	○	○	○	○	○
I feel I belong in chemistry	○	○	○	○	○
I feel I belong in STEM	○	○	○	○	○

	Frequency					Comfort				
Lecture	○	○	○	○	○	○	○	○	○	○
STEMentors	○	○	○	○	○	○	○	○	○	○
Groupwork	○	○	○	○	○	○	○	○	○	○
Instructor OH	○	○	○	○	○	○	○	○	○	○
TA OH	○	○	○	○	○	○	○	○	○	○
Email instructor	○	○	○	○	○	○	○	○	○	○
Email TA	○	○	○	○	○	○	○	○	○	○
Private tutoring	○	○	○	○	○	○	○	○	○	○
On-campus tutoring	○	○	○	○	○	○	○	○	○	○

Statement	SA	A	N	D	SD
I feel confident in chemistry	○	○	○	○	○
I have comparable skills to peers	○	○	○	○	○
I can apply concepts to problems	○	○	○	○	○
I understand units well	○	○	○	○	○
I use algebra effectively	○	○	○	○	○
Chemistry is useful for my goals	○	○	○	○	○
I feel I belong in chemistry	○	○	○	○	○
I feel I belong in STEM	○	○	○	○	○
I feel supported by teaching team	○	○	○	○	○
I work well in peer groups	○	○	○	○	○

What questions have you learned to ask yourself when stuck?

Have you changed how you interact with the AI tutor over time?

Advice for future students using the AI tutor:

Future use of generative AI in STEM courses:

Open Feedback
